# Pan-cancer analysis of ARFs family and ARF5 promoted the progression of hepatocellular carcinoma

**DOI:** 10.1016/j.heliyon.2024.e29099

**Published:** 2024-04-06

**Authors:** Qian Li, Fang Li, Xinqiu Song, Ning Lu, Xintao Jing, Hua Wen, Peihan Ma, Hua Zhang, Wenzhu Yao, Xiaofei Wang, Mingxin Zhang

**Affiliations:** a The First Affiliated Hospital of Xi'an Medical University, Xi'an, 710077, Shaanxi, China; b Institute of Genetics and Development Biology, School of Basic Medical Sciences, Xi'an Jiaotong University, Xi'an, 710061, Shaanxi, China; c Medical College of Yan'an University, Yan'an, 716000, Shaanxi, China; d Shaanxi University of Traditional Chinese Medicine, Xianyang, 712046, Shaanxi, China; e Xi'an Medical University, Xi'an, 710021, Shaanxi, China; f Biomedical Experimental Center, Xi'an Jiaotong University, Xi'an, 710061, Shaanxi, China

**Keywords:** Pan-cancer, ARFs, ARF5, Hepatocellular carcinoma, WGCNA

## Abstract

**Background:**

ARF family proteins are a kind of small GTPases, which are involved in regulating a variety of basic functions of cells. In recent years, the role and molecular regulatory mechanisms of ARFs in tumor progression have received increasing attention, and research reports on most of their family members are increasing. However, research on the clinical and pathological relevance of ARF5 in cancer, especially in hepatocellular carcinoma, still needs to be improved.

**Methods:**

RNA-seq data in the Cancer Genome Atlas (TCGA) and genome tissue expression (GTEx) databases were used to analyze the expression and pathological data of ARFs family in Pan-cancer. Kaplan-Meier and Cox regression were used for prognostic analysis of ARF5 and Pan-cancer. Combined with ImmuCellAI database and TIMER2 database, the relationship between ARF5 expression and immune cell tumor infiltration in hepatocellular carcinoma (HCC) was analyzed. WGCNA is used to construct the co-expression gene network related to ARF5 expression in HCC and screen important modules and central genes. GO and KEGG path enrichment analysis were carried out for the genes in the modules with clinical significance. GSEA analysis was performed to take into account the role of genes with small differences. Finally, ceRNA network analysis was used to explore the molecular mechanism of miRNAs and lncRNAs regulating ARF5 expression.

**Results:**

ARFs family (ARF1, ARF3, ARF4, ARF5, ARF6) are generally highly expressed in Pan-cancer. ARF5 is significantly highly expressed in 29 cancers, and the high expression of ARF5 in HCC patients is significantly negatively correlated with OS, DFI, PFI and DSS, which may lead to cancer deterioration by participating in tumor immune infiltration of HCC. Through WGCNA analysis, the expression of ARF5 in HCC may be involved in many cellular processes that consume a lot of energy, such as ribosome formation, RNA and protein synthesis and lipids, as well as COVID-19, nonalcoholic fatty liver, neurodegenerative diseases and other disease pathways.

**Conclusion:**

ARFs, especially ARF5, are overexpressed in many human tumors. This study shows for the first time that ARF5 is significantly correlated with the poor prognosis of HCC patients, which may play a role as an oncogene, suggesting that ARF5 has the potential as a biomarker for the diagnosis and treatment of HCC.

## 1 Introduction

Cancer is still a major disease that cannot be completely conquered and the main cause of death. It is estimated that there will be 19.3 million new cancer cases and nearly 10 million cancer deaths worldwide in 2020 [1], and the global cancer burden is expected to be 28.4 million in 2040, an increase of 47 % year-on-year in 2020 [2]. Therefore, further research and improvement of molecular regulation mechanisms of cancer occurrence and development, developing new tumor molecular markers, especially broad-spectrum biomarkers with significant significance in different cancers, remains the top priority and great challenge of medical research [3,4].

The protein encoded by the human ADP-ribosylation factor (ARFs) gene family is a small GTPase with a size of about 20 kDa [5]. Like Ras protein superfamily, ARF proteins acts on G protein A subunit as a cofactor of ADP ribotransferase by regulating the switching between the active GTP binding conformation and inactive GDP binding conformation [6,7]. The ARFs gene family encodes five ARF proteins, which are divided into class I (ARF1 and ARF3), class II (ARF4 and ARF5) and class III (ARF6) [8]. ARFs play an important role in intracellular material transport, cell signal transduction, cell membrane and lysosomal journey [9]. In recent years, the role and molecular mechanism of ARFs in tumors have gradually attracted the attention of researchers. The high expression of ARF1 will lead to the proliferation, migration and invasion of gastric cancer cells, and is related to the poor prognosis of patients [10]，and there are currently studies on anticancer drugs targeting ARF1 and its downstream regulatory molecules in colorectal cancer and breast cancer [11,12]. ARF6 regulates cancer progression by activating cell movement and invasion, and can promote the cell biological behavior of prostate cancer and liver cancer [13,14]. There have been articles summarizing the comprehensive functions of ARF6 in future cancer treatment, including ARF6-GEF, protein structure, and its role in cancer [15]. ARF4 has also been studied in many cancers, such as ARF4 promotes the proliferation and migration of epithelial ovarian cancer cells and can be regulated by miR-221-3p [16], and there are studies reporting that ARF4 may serve as a novel serum autoantibody biomarker for early diagnosis of gastric cancer and precancerous lesions [17]. However, there are still few reports on the biological functions and molecular regulatory mechanisms of ARF5 in cancer, which needs to be filled in to explore the potential value of ARF5 in cancer screening and treatment. Although there are relatively few articles in this regard, the current findings support ARFs's cancer promoting role, suggesting that its family molecules may be a potential biomarker for cancer. There is currently no comprehensive report on the potential expression of the ARFs gene family in pan-cancer, as well as the clinical relevance and possible molecular regulatory mechanisms of ARF5 in cancer, especially in hepatocellular carcinoma (HCC).

In this study, we explored the expression of ARFs gene family in Pan-cancer as the starting point, selected the most closely related ARF5 in cancer development, and further screened out the most closely related HCC with ARF5. Then we analyzed the clinical and pathological correlation between ARF5 and HCC, and finally explored the role of ARF5 in HCC and its potential molecular network regulatory mechanisms. The conclusions of this study may provide a certain research foundation and new ideas for future cancer related research on ARF5.

## 2 Materials and methods

### 2.1 ARFs family expression in pan-cancer

The expression profiles of Pan-cancer ARFs were downloaded from the RNA-seq dataset of the Cancer Genome Atlas database (TCGA, https://xena.ucsc.edu), while the RNA-seq dataset of normal control tissues was collected from genotype tissue expression (GTEx, https://commonfund.nih.gov/GTEx/). All the obtained data were first preprocessed to remove low-quality data and missing values, followed by Z-score standardization and batch effect correction, and subsequent data analysis. The specific information of all gene expression is summarized in Table S1. The RNA-seq data sets of TCGA and GTEx counts were standardized, then the ggpubr package of the R software (version 4.2.2, http://www.r-project.org/) [18] was used for statistical analysis, and the boxplot of gene expression was drawn by ggplot2 package [19] of the R software. Non paired student t-test (assuming homogeneity of variance) is used to evaluate expression differences between adjacent control tissues and cancer tissues. Significance level (α) is set to 0.05.

### 2.2 Prognostic analysis of ARF5 in pan-cancer

Kaplan-Meier analysis [20] was used to evaluate the correlation between the expression of ARF5 downloaded from TCGA database in 33 cancers and the corresponding prognosis. The expression values were log2 transformed, and the cancer patients were divided into high expression group and low expression group by using the surv_cutpoint function of the survminer. Then univariate Cox regression analysis was performed to draw the Kaplan-Meier survival curves of overall survival rate (OS), disease-free interval rate (DFI), disease-specific survival rate (DSS) and progression-free survival rate (PFI) of ARF5 in Pan-cancer.

### 2.3 Expression correlation of ARFs

According to the gene expression level calculated by TCGA database, the co-expression relationship between ARF family genes was analyzed by Pearson correlation analysis using R corrplot package.

### 2.4 Correlation between ARF5 and immune cell infiltration

The Human Protein Atlas database (https://www.proteinatlas.org/) was used to analyze the expression of ARF5 in all types of cells contained in human liver tissue [21]. The immune cell infiltration fraction was downloaded from the ImmuCellAI database (http://bioinfo.life.hust.edu.cn/web/ImmuCellAI/) and TIMER2 database (http://timer.cistrome.org/) [22,23], which for tumor immune evaluation resources, including B cells, CD4+T cells, CD8^+^ T cells, neutrophils, macrophages and myeloid dendritic cells. Then, Spearman correlation analysis was carried out based on the log2 of ARF5 expression in TCGA database to evaluate the correlation between ARF5 and tumor infiltrating lymphocyte marker genes.

### 2.5 Data normalization and recognition of DEGs

Robust multi array averaging (RMA) method is used to preprocess and normalize the database. The differences between samples were analyzed by using the limma software package, and multiple hypothesis tests and corrections were performed after obtaining the p value. The threshold of P value is determined by controlling the error detection rate (FDR), and the corrected p value (Q value) is adjusted [24]. The screening criteria were log2 (fold change) > 1 or < −1 and the adjusted p value (Q value) < 0.05. We corrected the p value of FDR in the following KEGG analysis, GO analysis and GSEA analysis.

### 2.6 WGCNA analysis of ARF5 in HCC

The R package of WGCNA (weighted gene co-expression network analysis) is used to identify the differentially expressed genes related to ARF5 in HCC, analyze the relationship between gene set and ARF5 expression, draw the regulatory network between genes in the gene set and screen out key regulatory genes [25]. Firstly, 25 % of the variance of DEGs is selected, outliers are removed, and a similarity matrix is constructed using expression data. The Pearson correlation coefficient is used to calculate the absolute value of the correlation coefficient between pairs of genes, which is transformed into a Pearson correlation matrix. Then, the similarity matrix is transformed into an adjacency matrix. Next, the adjacency matrix is transformed into a topological overlap matrix (TOM) to describe the association strength between genes. Finally, TOM is used as the input of gene hierarchical cluster analysis, and the module tree is drawn [26].

### 2.7 Identification of DEGs and enrichment analysis

GO (Gene ontology) analysis and KEGG pathway analysis of differentially expressed genes in WGCNA were performed using David 6 online tool (https://david-d.ncifcrf.gov/) [27,28]. GO analysis can enrich differential genes of biological process (BP), cellular component (CC), and molecular function (MF). During enrichment, the unique GO-ID number can be obtained through the enrichment of differential genes, and then the genes can be enriched into different pathways. KEGG mainly focuses on signal pathways. KEGG database can enrich different genes in different signal pathways, there are often many pathways that can be enriched, so we choose the pathway with lower p-value to explain [29].

### 2.8 Gene set enrichment analysis (GSEA)

GSEA [30] is an enrichment analysis method based on gene set. When analyzing gene expression data, first determine the purpose of analysis, that is, select one or more functional gene sets in MSigDB for analysis, and then sort the size based on the changes of gene expression data and expression amount. Then judge whether the genes in each gene set are enriched in the upper or lower part of the gene list after phenotypic correlation ranking, to judge the influence of the synergistic change of genes in this gene set on the phenotypic change [30]. GSEA finds out the gene sets with concordant differences from the expression matrix of all genes, so it can take into account the genes with relatively small differences.

### 2.9 Construction of ceRNA network of ARF5 in HCC

TCGA database was used to analyze the gene expression profiles of lncRNA and miRNA related to ARF5 expression in liver cancer, and edgeR software package was used to analyze the high expression genes related to ARF5 (the correlation of lncRNA and ARF5 is greater than 2 [31], and the correlation of miRNA and ARF5 is less than −2). Subsequently, gene names were annotated using Gencode (https://www.gencodegenes.org/) to obtain the lncRNA differential gene matrix [32]. The miRcode website (http://mircode.org/) is used to predict the miRNA family targeting the 3′UTR region of ARF5 and lncRNAs targeting the above miRNAs [33]. Then intersect the predicted miRNA with the miRNA negatively correlated with ARF5, and intersect the predicted lncRNA with the highly expressed lncRNA to obtain the corresponding miRNA and lncRNA set. Finally, the data were and loaded into Cytoscape (vision 3.7.1) to obtain the ceRNA network [34].

### 2.10 Cell culture

We purchased human liver cancer cell lines MHCC-97H, Huh7, and normal liver cell line HL-7702 from Zhongqiao Xinzhou Biotechnology Co., Ltd. (Shanghai, China) and grown in a constant temperature incubator at 37 °C and 5 % CO^2^. Cells were cultured in RPMI1640 medium containing 10 % fetal bovine serum [35].

### 2.11 RNA extraction and real-time quantitative PCR (qRT-PCR)

Use TRIzol reagent (Sangon Biotech, Shanghai, China) to degrade cells and isolate total RNA. According to the manufacturer's instructions (Sangon Biotech), cDNA was synthesized by reverse transcription, and qRT-PCR was performed using the SYBR Green PCR kit (Sangon Biotech). Standardize gene expression using GAPDH as an internal reference. All primers used in this study: ARF5-F: 5′-GCAGATGCGGATTCTCATGG-3′; ARF5-R: 5′-AAAGATGAGGCCCTGAGTGT-3′; GAPDH-F: 5′-GGAGCGAGATCCCTCCAAAAT-3′ [36]; GAPDH-R: 5′-GGCTGTTGTCATACTTCTCATGG-3′. Use 2^−ΔΔCT^ method calculates the relative fold change in RNA expression.

## 3 Results

### 3.1 ARFs families are significantly overexpressed in pan-cancer

**Fig. 1:**
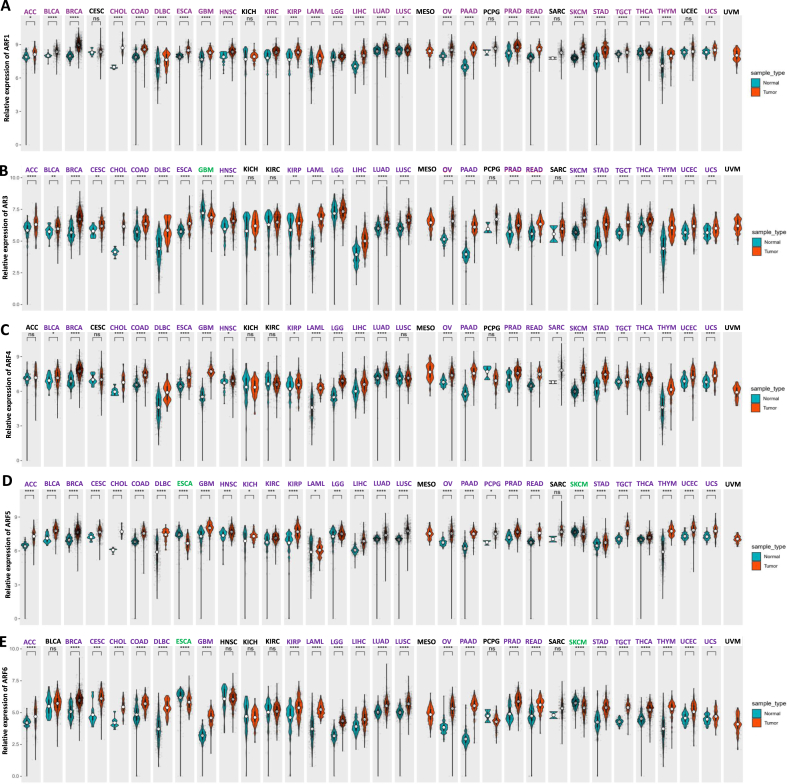
**The expression levels of ARFs gene family in 33 Pan-cancer were analyzed by TCGA and GTEx databases.** ARF1 (A), ARF3 (B), ARF4 (C), ARF5 (D), ARF6 (E) are highly expressed in 26, 26, 26, 28, and 23 types of cancers, respectively. The blue group in the figure represents the gene expression in normal tissues, while the orange group represents the gene expression in cancer tissues; The larger the a rea of the color area in the group, the greater the probability of distribution near the value; The black part in the figure is a box plot, and the white circle represents the median. Purple character represents significantly high expression, and green character represents significantly low expression.

Based on the role of small GTPase of ARFs family and its important functions in basic cellular processes such as material transport, signal transduction and cell formation, we focused on the regulation of ARFs on human cancer. We first compared the expression levels of ARFs families in 33 cancers and their corresponding normal control tissues in GTEx and TCGA databases (Fig. 1), and the calculated specific expression values are shown in Table S1. Except for those cancers without normal tissue data (mesothelioma and uveal melanoma), the results showed that ARFs family is highly expressed in most cancer types. Among them, ARF1 was significantly overexpressed in 26 cancers (Fig. 1A); ARF3 was significantly overexpressed in 26 cancers and only low in glioblastoma multiforme (Fig. 1B); ARF4 was significantly overexpressed in 26 cancers (Fig. 1C); ARF5 was significantly overexpressed in 28 cancers and only low expressed in esophageal carcinoma (Fig. 1D); ARF6 was significantly overexpressed in 23 cancers, while it was low expressed in esophageal carcinoma and skin cutaneous melanoma (Fig. 1E). This shows that ARFs family is a key molecular family to regulate cancer progression. The cancer promoting effects of ARF1, ARF3, ARF4 and ARF6 in individual cancers have been studied, only the role of ARF5 in cancer has not been reported. Therefore, we will focus on ARF5 gene to explore the relationship between its expression and cancer and its potential regulatory pathways.

### 3.2 High expression of ARF5 in HCC is associated with poor prognosis and low survival rate

**Fig. 2:**
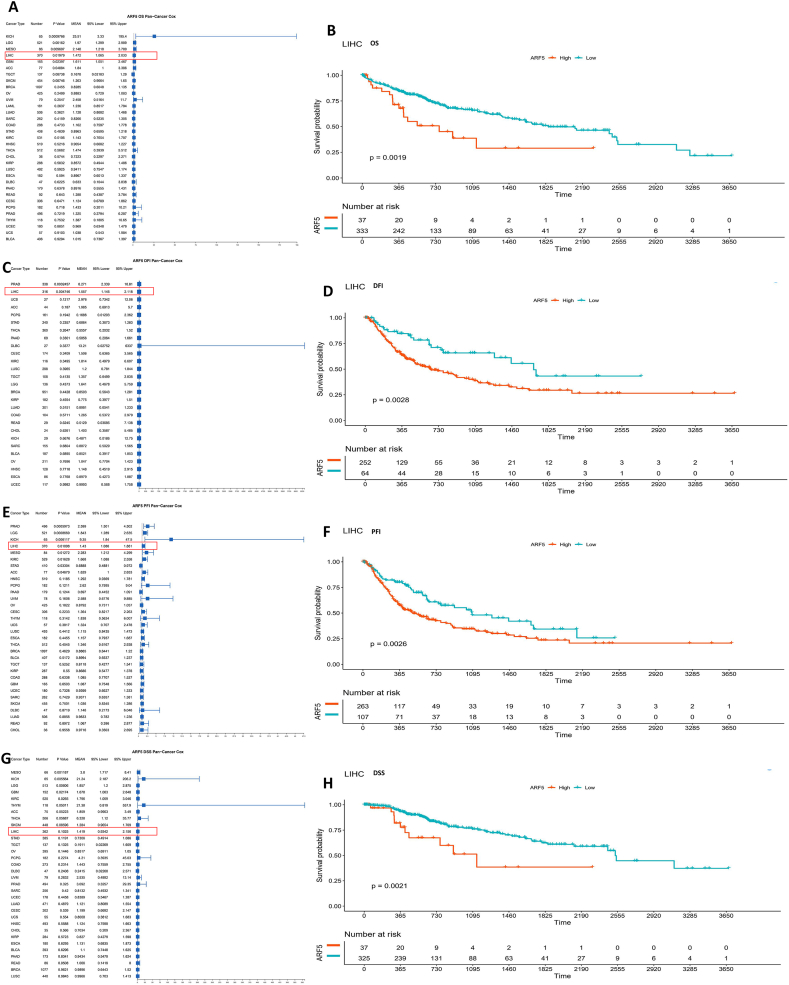
**Relationship between ARF5 expression and OS, DFI, PFI and DSS of Pan-cancer.** The forest plot showed the correlation between ARF5 expression and OS (A), DFI (C), PFI (E) and DSS (G) of various tumor types in TCGA. Kaplan Meier curves calculated the prognostic correlation of OS (B), DFI (D), PFI (F) and DSS (H) between low expression group and high expression group of ARF5 in HCC. In the forest map, a vertical line with a horizontal scale of 1 represents the invalid line, a horizontal line for each group represents the 95 % confidence interval, and the area of the blue box represents the weight. Overall, the shorter the confidence interval, the greater the weight, and the higher the credibility of the results. In the Cox proportional risk model, Sort the expression of ARF5 in cancer tissue from low to high, with the first 50 % being the low expression group and the last 50 % being the high expression group. Further plot the Kaplan Meier curves. The blue line represents low expression of ARF5, the red line represents high expression of ARF5, the x-axis represents survival days, the y-axis represents survival likelihood, and the values in the table represent specific numbers of people. The red line is below the blue line, indicating that the higher the ARF5 expression, the lower the survival likelihood.

To investigate the potential prognostic value of upregulation of ARF5 in Pan-cancer, we divided the ARF5 gene expression profile of 33 tumor samples from TCGA into high expression group and low expression group, determining the relationship between ARF5 expression and overall survival rate (OS) and disease free interval rate (DFI) through Cox proportional hazards model analysis, and drew the survival forest map and K-M survival plots (Fig. 2A and. C, Figure S1A, Figure S1B). Among them, about half of the patients with upregulated expression had worse prognosis of OS and DFI than those with low expression (*P* < 0.05). Considering that there may be other factors other than tumor death during follow-up, we also compared the progression-free interval (PFI) and disease-specific survival (DSS) (Fig. 2E and. G, Figure S1C, Figure S1D). The results are not surprising that the high expression of ARF5 predicts poor PFI and DSS in half of the cancers. Based on the above results, the high expression of ARF5 is significantly negatively correlated with OS, DFI, PFI and DSS in adrenocortical carcinoma (ACC), brain lower grade glioma (LGG) and liver hepatic carcinoma (LIHC), and LIHC contains more patients (Fig. 2B–. D, Fig. 2F–. H). Therefore, we will focus on the expression of ARF5 in hepatocellular carcinoma and its influence mechanism on tumor progression. In addition, we also analyzed the expression of ARF5 mRNA in human liver cells HL-7702 and liver cancer cell lines MHCC-97 and Huh7 (Fig. S2). The results showed that the expression of ARF5 in MHCC-97 and Huh7 was significantly higher than that in HL-7702, which to some extent suggests that ARF5 may play a role as an oncogene in HCC.

Next, we considered whether the expression of ARFs in HCC was consistent (Fig. S3). In TCGA database, the expression of ARF5 and other members of ARFs in tumor tissues and normal tissues has a very significant positive correlation, indicating that the biological function and molecular regulation mechanism of ARF5 in HCC maybe have a certain reference value for other members of ARFs.

### 3.3 ARF5 is involved in the immune cell tumor infiltration of HCC

**Fig. 3:**
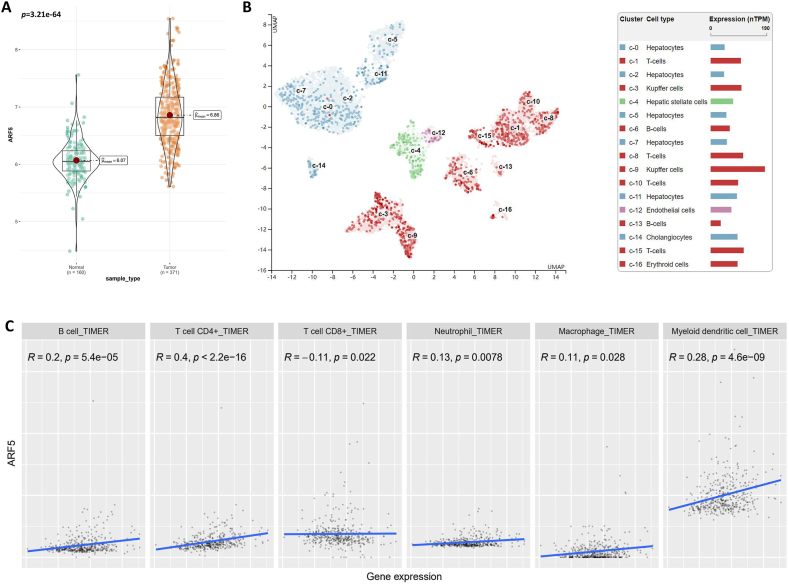
**Relationship between ARF5 expression and tumor infiltration of different immune cells in HCC. (A)** The expression levels of ARF5 in 371 HCC tissues and 160 normal tissues which analyzed by TCGA and GTEx databases. **(B)** The expression of ARF5 in various types of cells in human liver tissue which analyzed by the human protein atlas database. **(C)** ImmuCellAI database and TIMER2 database evaluated the correlation between ARF5 expression and tumor infiltrating lymphocyte marker genes in HCC.

The joint analysis of TCGA and GTEx databases showed that the expression of ARF5 in 371 HCC tissues was significantly higher than that in 160 normal control tissues (Fig. 3A). Through the Human Protein Atlas database, we learned about the expression of ARF5 in liver cells and related immune cells in human hepatocellular carcinoma tissues, and to determine the relationship between ARF5 and tumor immune microenvironment. The analysis results showed that the expression of ARF5 in immune-related cells such as T cells and B cells was higher than that in liver cells (Fig. 3B), suggesting that the expression of ARF5 may be related to immunity. Therefore, we further investigated the relationship between the expression of ARF5 and the level of related immune cell infiltration in hepatocellular carcinoma (Fig. 3C). Our data showed that in the correlation with immune cells, the expression of ARF5 has a relatively good positive correlation with CD4+T cells (R = 0.4, *p* < 2.2e-16), while it has a relatively weak correlation with other types of immune cells such as invasive memory B cells (R = 0.2, *p* = 5.4e-05), neutrophils (R = 0.13, *p* = 0.0078), macrophases (R = 0.11, *p* = 0.028), Myeloid dendritic cell (R = 0.28, *p* = 4.6e-9), and CD8+T cells (R = −0.11, *p* = 0.022). This suggested that ARF5 might participate in the infiltration process of immune cells by promoting the activation of CD4+T cells, promoting immune escape of tumor cells, thereby promoting the progression of HCC and leading to malignant prognosis.

In addition, we also made further analysis on the relationship between the expression of ARF5 and immune cells in Pan-cancer (Fig. S4). The results show that ARF5 does have a significant correlation with immune cells in most cancers, suggesting that ARF5 plays an important role in Pan-cancer.

### 3.4 ARF5-related expression gene network in HCC

**Fig. 4:**
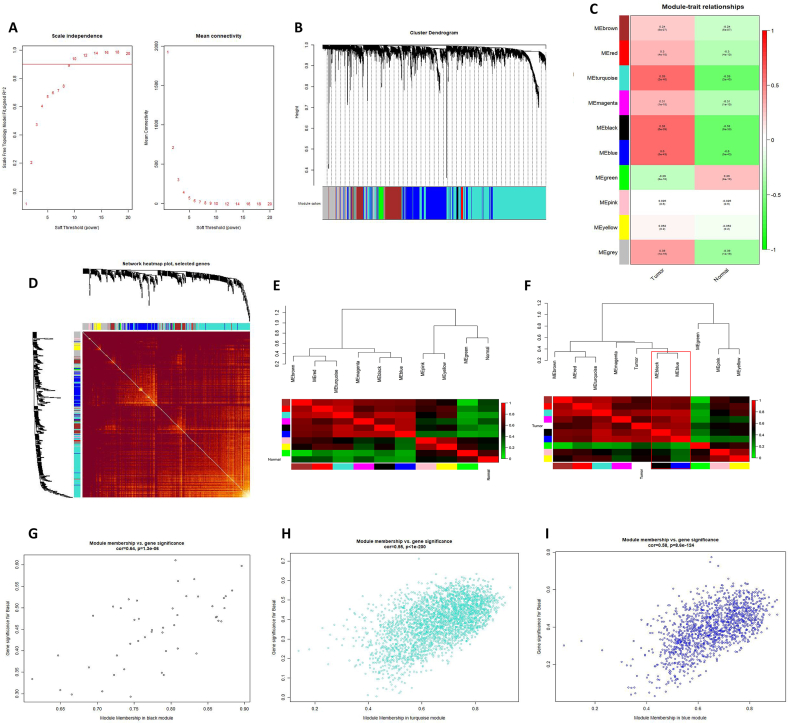
**WGCNA analysis of ARF5 expression in HCC. (A)** Calculation and determination of optimum soft threshold power. The left figure represents the influence of different powers on scale independence, and the x-axis reflects the soft threshold capability, Y-axis reflects the fitting index of scale-free topological model; The figure on the right represents the influence of the same power on the average connectivity. The x-axis reflects the soft threshold capability and the y-axis reflects the average connectivity. The red line indicates the selected power threshold. **(B)** Clustering tree of data sets composed of differentially expressed genes. The genes in the module are marked with different colors. Gray indicates that there are no genes in any module. **(C)** Association diagram of module genes and characteristics. Each row corresponds to a module gene, and each column corresponds to a feature (including tumor and normal). The number in the first row of each cell corresponds to the correlation coefficient of gene and feature, the cell color is colored according to the correlation, red represents positive correlation, green represents negative correlation. The number in brackets in the second row corresponds to *p* value. **(D)** The heat map shows the topological overlap matrix (TOM) of all DEGs. The darker the color, the higher the overlap. The gene tree is displayed on the left and top. **(E**–**F)** Eigengene dendrogram and eigengene adjacency plot. The heat map shows the hierarchical clustering tree of module characteristic genes and microarray sample characteristics y and the heat map of adjacent parts in the characteristic gene network. Each row corresponds to a module characteristic gene, and the column corresponds to the trait (normal is E, tumor is F). The cell color represents the adjacency relationship, red represents high adjacency (positive correlation), and green represents low adjacency (negative correlation). **(G**–**I)** The relationship between different color module membership and tumor gene significance. The genes of black module (G), turquoise module (H) and blue module (I) are 47, 2767 and 1368 respectively.

In order to explore the co-expression network of ARF5 in HCC, we performed WGCNA analysis. Firstly, we calculated the soft thresholding power β and proposed the co-expression similarity to calculate the adjacency degree (Fig. 4A), constructed the gene network and identified the modules using WGCNA R software package (Fig. 4B and C), and carried out the topological overlap matrix (TOM) for the relationship between the identified modules to show that the gene expression is relatively independent between modules (Fig. 4D), the genetic information in each module is shown in Table S2. Then we analyzed the connectivity of characteristic genes and clustered the modules according to the expression in normal and tumor tissues (Fig. 4E and F). The results showed that among the 10 modules analyzed, the turquoise module, black module and blue module had the highest significant correlation with tumor. After analyzing the correlation between the genes in these three modules and ARF5 expression (Fig. 4G–I), we selected the blue module with the higher correlation with HCC and gene significance of ARF5 expression and more genes for further biological function network analysis.

### 3.5 Functional analysis of the blue module

**Fig. 5:**
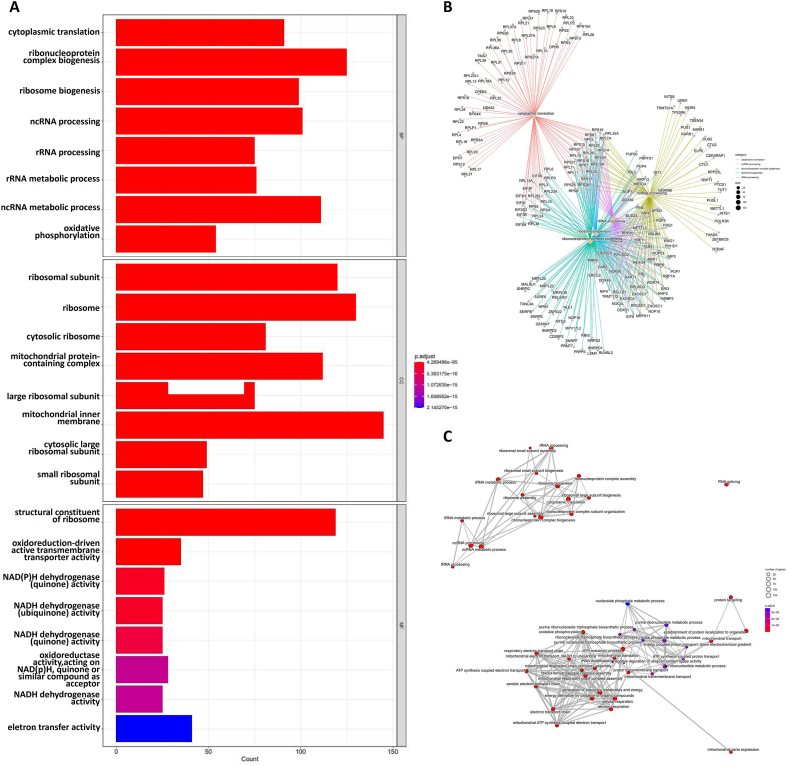
**Gene ontology analysis of biological processes, cell components and molecular functions based on DEGs in blue module.** (A) The barplot of the DEGs in blue module. The figure shows the top eight functional terms in BP, CC and MF. (B) The centplot of the DEGs in blue module which shows the inclusion relationship between the first five enrichment terms of BP and genes. (C) The emapplot of the DEGs in blue module which shows the common gene relationship between the enrichment functional terms.

**Fig. 6:**
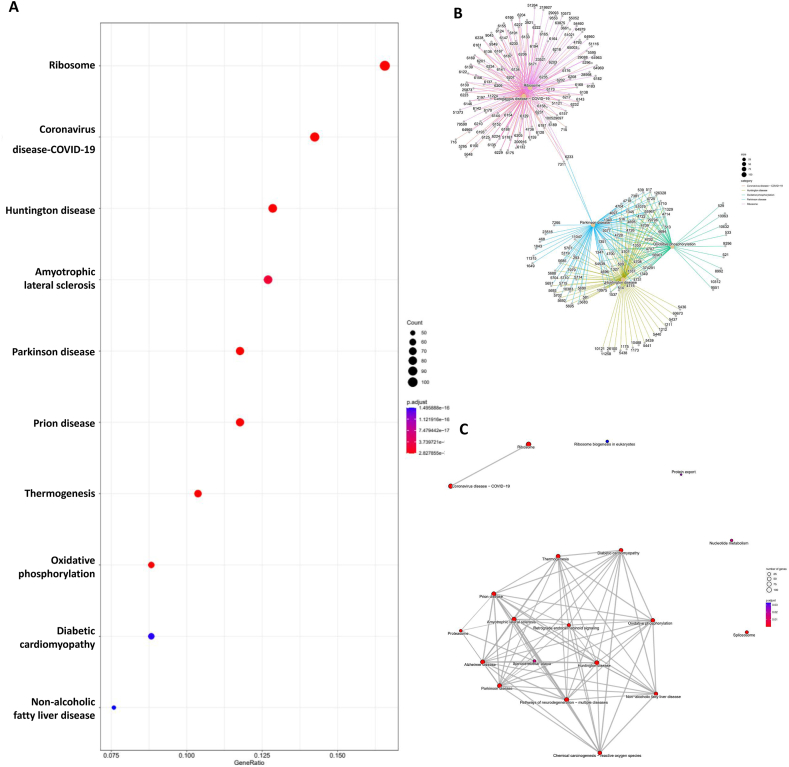
**KEGG analysis based on DEGs in blue module.** (A) The dotplot of the DEGs in blue module. The figure shows the top 20 functional pathways. The length of the column represents the number of genes, and the darker the red indicates the smaller the *p* value. (B) The centplot of the DEGs in blue module which shows the inclusion relationship between the first five enrichment pathways and genes. (C) The emapplot of the DEGs in blue module which shows the common gene relationship between the enrichment functional pathways.

After eliminating the genes with insignificant differences, we submitted 1348 target genes with significant differences in the blue module of omicshare to determine the representative GO term and KEGG pathways to further clarify their functional characteristics. The specific information of differential genes included in GO and KEGG analysis is shown in Table S3, and the information of all GO term and KEGG pathway analysis is shown in Table S4 and Table S5. Fig. 5A shows the eight most significant terms in the GO enrichment analysis of differentially expressed genes (DEGs), and shows the gene inclusion relationship between the first five terms in biological processes (Fig. 5B) and the correlation of all differential terms (Fig. 5C). The results showed that the DEGs associated with ARF5 expression were mainly involved in ribosome composition and mitochondrial intima formation, and played an important role in biological functions such as RNA and protein synthesis, material transport, energy consumption and transformation. Fig. 6A shows the KEGG enrichment analysis results of DEGs, and the molecular intersection and pathway correlation between pathways are shown in Fig. 6B and C. The results show that these DEGs related pathways are enriched not only in the ribosome and oxidative phosphorylation metabolism corresponding to GO analysis, but also in Coronavirus disease of COVID-19, neurodegenerative diseases, and non-alcoholic fatty liver disease. These data support that ARF5 plays a role in regulating the progression of liver disease to tumors, and the initial ARF5 may participate in the disease process of COVID-19 and nervous system diseases.

### 3.6 GSEA analysis of the blue module

**Fig. 7:**
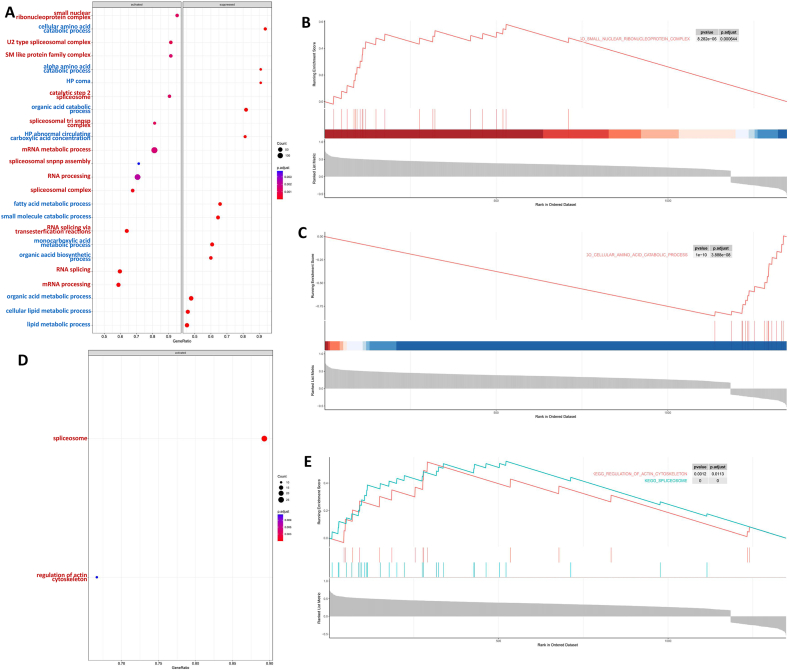
**Gene set enrichment analysis based on DEGs in the blue module.** (A) The barplot of GO enrichment analysis for genes co-expressed with ARF5 in blue module based on GSEA. Red represents pathways positively correlated with ARF5 expression, while blue represents pathways negatively correlated. (B) GO analysis of broken line of gene set with the highest positive correlation with ARF5.The horizontal axis is each gene under the gene, and the vertical axis is the corresponding Running ES, and the value greater than zero indicates that the gene set is positively correlated with ARF5. The peak in the broken line graph is the Enrichemnt Score of the gene set, and the gene before the peak is the core gene under the gene set. (C) GO analysis of broken line of gene set with the highest negative correlation with ARF5. (D) The dotplot of KEGG enrichment analysis for genes co-expressed with ARF5 in blue module based on GSEA. (E) KEGG analysis of broken lines of gene sets positively associated with ARF5.

We also used GSEA to analyze the gene set of blue module to more accurately determine the function of hub genes, which can take into account the genes with small differences, and in pathway analysis, focus on metabolic pathways and molecular interaction networks. In GO enrichment analysis of GSEA, the pathways positively related to ARF5 expression are mainly RNA synthesis and translation processes and related splice complexes, while the pathways negatively related are mostly related to amino acid and lipid metabolism (Fig. 7A–C). Probably due to the algorithm of GSEA analysis, KEGG pathway positively related to ARF5 is mainly in two energy consuming pathways: the formation of spliceosomes and the regulation of actin cytoskeleton (Fig. 7D and E).

### 3.7 miR-29 and its associated lncRNA may target and regulate the expression of ARF5 in HCC

**Fig. 8:**
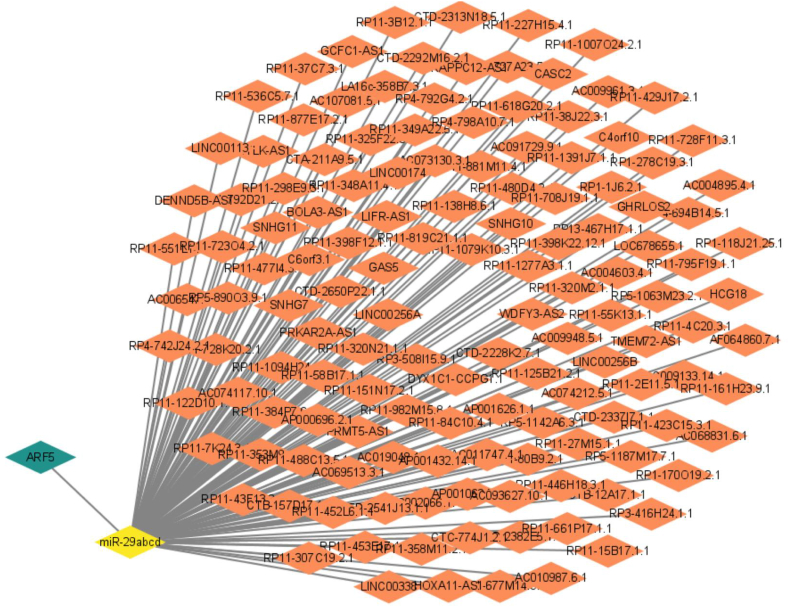
Bioinformatics database analysis of the ceRNA network involved in the regulation of ARF5 expression in HCC.

Finally, we considered exploring the noncoding RNA regulating ARF5 expression. ceRNA includes lncRNA, circRNA and mRNA, and they can bind to miRNA through microRNA response elements (MRes) to inhibit miRNA mediated gene silencing. Through TCGA database and bioinformatics analysis of miRNAs targeted to ARF5, we screened that miR-29 may inhibit the expression of ARF5. Combined with the analysis of ceRNA targeted to miR-29 and lncRNA positively related to the expression of ARF5, we screened 138 ceRNAs, which may promote the expression of ARF5 in HCC through competitive binding of miR-29. The ceRNA network involved in ARF5 regulation in HCC is shown in Fig. 8 and Table S6.

## 4 Discussion

Primary liver cancer is the fifth most common cancer and the third leading cause of death in the world [37], and hepatocellular carcinoma (HCC) accounts for more than 85–90 % of primary liver cancer [38]. Due to the rapid growth of liver cancer, high postoperative recurrence rate and poor prognosis, the overall survival rate of patients is significantly lower than that of other common cancers, which seriously threatens human life and health [39]. Therefore, it is of great research value to explore the biomarkers of HCC in the process of early disease progression and clarify the molecular regulatory network of signal transduction [40]. In this study, we analyzed the relationship between the expression and prognosis of ARF family members in Pan-cancer through bioinformatics database. It was found that all ARFs were highly expressed in more than 2/3 cancers and correlated with the poor prognosis of 1/2 cancer patients. After further analysis, we found that the high expression of ARF5 was significantly negatively correlated with the survival time of HCC patients such as OS, DFI, PFI and DSS, indicating that ARF5 is a cancer biomarker with great potential research value.

ARF5 belongs to ARFs family and is a small GTPase, which are a large group of hydrolases with a highly specific G domains, and can bind and hydrolyze GTP. GTPase plays an important role in many important signal transduction pathways controlling cell growth, migration and differentiation [41]. ARF5 mainly locates in the middle region of the endoplasmic reticulum/Golgi and CIS Golgi, and is involved in regulating the transport from endoplasmic reticulum to Golgi [42]. Recent studies have found that ARF5 is more widely distributed on the plasma membrane and can also be observed in small spots co-located with clathrin [43]. Studies have shown that ARFs family proteins are related to membrane transport and signal mechanisms of cell invasion. ARF GTPases play a basic role in vesicle transport by regulating ribosomal synthesis and transport complexes, lipid modifying enzyme assembly, and recruiting other regulatory factors of GTPase to the damaged membrane [43]. Through WGCNA analysis and GSEA analysis, our study also confirmed that in HCC, the signal pathways enriched by the gene co-expression network related to ARF5 expression are basically ribosome composition, mitochondrial intima and material transport, suggesting that ARF5 provides the required energy and material in the process of malignant transformation of hepatocytes and plays an important role in the progression of HCC.

In terms of mechanism, reports on the impact of ARFs on signal transduction have been accumulating [44]. Due to its role as a regulatory GTP enzyme, the most studied cellular function of ARFs is to control membrane transport and actin remodeling [45,46], which can affect signal transduction through various mechanisms, such as the transport of receptors or signaling cascade components and the cytoskeletal scaffold of signaling proteins [45,46]. The role of ARF in membrane transport and actin remodeling depends on the controlled binding and hydrolysis of GTP, which is regulated by guanine nucleotide exchange factors (GEFs) and GTP enzyme activating proteins (GAPs), respectively. Many ARF GEFs and ARF GAPs are structurally complex, and in addition to their catalytic domains, they also have the ability to bind to signal molecules other than ARFs and regulate their functional domains. Some ARF GAP and ARF GEF are associated with differentiation and carcinogenesis, as well as signal transduction interruption. In addition, although some ARF effectors are directly related to the role of ARF in membrane transport, other effectors control signaling molecules, such as phosphoinositol or Rho family proteins. Therefore, our study suggests that the correlation between ARF5 and ribosome composition, mitochondrial inner membrane, and substance transport pathways is likely related to its signal transduction, which is worth studying.

The link between immunity and cancer progression has always been the focus of medical research [47]. In recent years, immunotherapy for liver cancer has gradually become a new hotspot [48]. However, due to individual differences between patients, only a small number of people can benefit from immunotherapy [49]. As an indispensable part of immunotherapy, the tumor immune microenvironment (TIME) has gradually attracted people's attention. The analysis of time will help to improve the responsiveness of immunotherapy [50]. This is the first time that we have confirmed that there is a significant correlation between ARF5 expression and HCC related immune cell infiltration. In the related cells of hepatocellular carcinoma, the correlation between ARF5 expression and immune cells is significantly greater than that of hepatocytes, and the signaling pathway that may be regulated by ARF5 co-expression gene network is significantly related to nonalcoholic fatty liver, indicating that ARF5 may play an important role in the progression of nonalcoholic fatty liver to hepatocellular carcinoma, and may participate in the regulation and destruction of the immune microenvironment of liver tissue. It is suggested that ARF5 may be a potential target to regulate the immune microenvironment of HCC and eliminate the individual differences of immunotherapy.

It is worth mentioning that our analysis results show that ARF5 related coexpression gene network may also be involved in the diseases process of coronavirus disease 2019 (COVID-19). Although our current data are still relatively weak, this is also a discovery beyond our expectation, which also provides a direction for the current research on COVID-19.

However, although the research findings fully confirm the expression and potential role of ARF5 in HCC, and further suggest the upstream molecule miR-29 that regulates its expression, our research is currently only based on bioinformatics databases and bioinformatics analysis. Next, we will propose a more rigorous logic research and conduct experiments in vitro and in vivo to fully confirm the role of ARF5 in HCC and verify its specific mechanisms in energy transport and immune microenvironment.

## 5 Conclusions

In summary, we reported the high expression of ARFs family in most cancers, and further analyzed that the high expression of ARF5 leads to poor prognosis in hepatocellular carcinoma and may be involved in the regulation of the immune microenvironment of HCC. Through WGCNA and GSEA analysis, we further confirmed that ARF5 is involved in splicing complex, ribosome synthesis and function, intracellular material transport and other pathways, and is related to the progress of some neuropathy diseases, COVID-19, nonalcoholic fatty liver and other diseases. Finally, we analyzed the molecular mechanism of miR-29 targeting the 3′UTR region of ARF5 and inhibiting its expression, which needs to be verified by further research.

## Funding

Shaanxi Provincial Department of Science and Technology, China; Shaanxi Provincial Health Commission, China; Xi'an Association of Science and Technology, China; Xi'an Medical College China.

## Data availability statement

All data are included in article/supplementary material/referenced in article.

## CRediT authorship contribution statement

**Qian Li:** Writing – original draft, Visualization, Data curation, Conceptualization. **Fang Li:** Investigation, Formal analysis, Data curation. **Xinqiu Song:** Methodology, Data curation. **Ning Lu:** Software, Investigation. **Xintao Jing:** Project administration, Data curation. **Hua Wen:** Investigation, Data curation. **Peihan Ma:** Resources, Investigation. **Hua Zhang:** Visualization, Formal analysis. **Wenzhu Yao:** Writing – review & editing, Writing – original draft, Conceptualization. **Xiaofei Wang:** Writing – review & editing, Visualization, Software, Data curation. **Mingxin Zhang:** Writing – review & editing, Writing – original draft, Conceptualization.

## Declaration of competing interest

The authors declare that they have no known competing financial interests or personal relationships that could have appeared to influence the work reported in this paper.

## Abbreviations

HCC: hepatocellular carcinoma
ARF5: ADP-ribosylation factor 5
TCGA: The Cancer Genome Atlas
GTEx: Genotype-Tissue Expression
OS: Overall survival
DFI: Disease-free interval
DSS: Disease-specific survival
PFI: Progression-free interval
LIHC: Liver hepatocellular carcinoma
ACC: Adrenocortical carcinoma
BLCA: Bladder Urothelial Carcinoma
BRCA: Breast invasive carcinoma
CESC: Cervical squamous cell carcinoma and endocervical adenocarcinoma
CHOL: Cholangio carcinoma
COAD: Colon adenocarcinoma
ESCA: Esophageal carcinoma
GBM: Glioblastoma multiforme
HNSC: Head and Neck squamous cell carcinoma
KICH: Kidney Chromophobe
KIRC: Kidney renal clear cell carcinoma
KIRP: Kidney renal papillary cell carcinoma
LAML: Acute Myeloid Leukemia
LGG: Brain Lower Grade Glioma
LUAD: Lung adenocarcinoma
LUSC: Lung squamous cell carcinoma
OV: Ovarian serous cystadenocarcinoma
PAAD: Pancreatic adenocarcinoma
PRAD: Prostate adenocarcinoma
READ: Rectum adenocarcinoma
SKCM: Skin Cutaneous Melanoma
STAD: Stomach adenocarcinoma
TGCT: Testicular Germ Cell Tumors
THCA: Thyroid carcinoma
UCEC: Uterine Corpus Endometrial Carcinoma
UCSUterine Carcinosarcoma. GO: Uterine Carcinosarcoma. GOGene ontology
KEGG: Kyoto Encyclopedia of genes and genomes
WGCNA: Weighted gene co expression network analysis
GSEA: Gene Set Enrichment Analysis
ceRNA: Competing endogenouse RNA
DEGs: Differentially expressed genes
TOM: Topological overlap matrix
COVID-19: Coronavirus disease 2019.
