# Supplementary figures and images for: Pan-cancer analysis of ARFs family and ARF5 promoted the progression of hepatocellular carcinoma
Source: Heliyon. 2024 Apr 6;10(7):e29099. doi: 10.1016/j.heliyon.2024.e29099 (PMC11015141; doi:10.1016/j.heliyon.2024.e29099)

**A****OS of ARF5**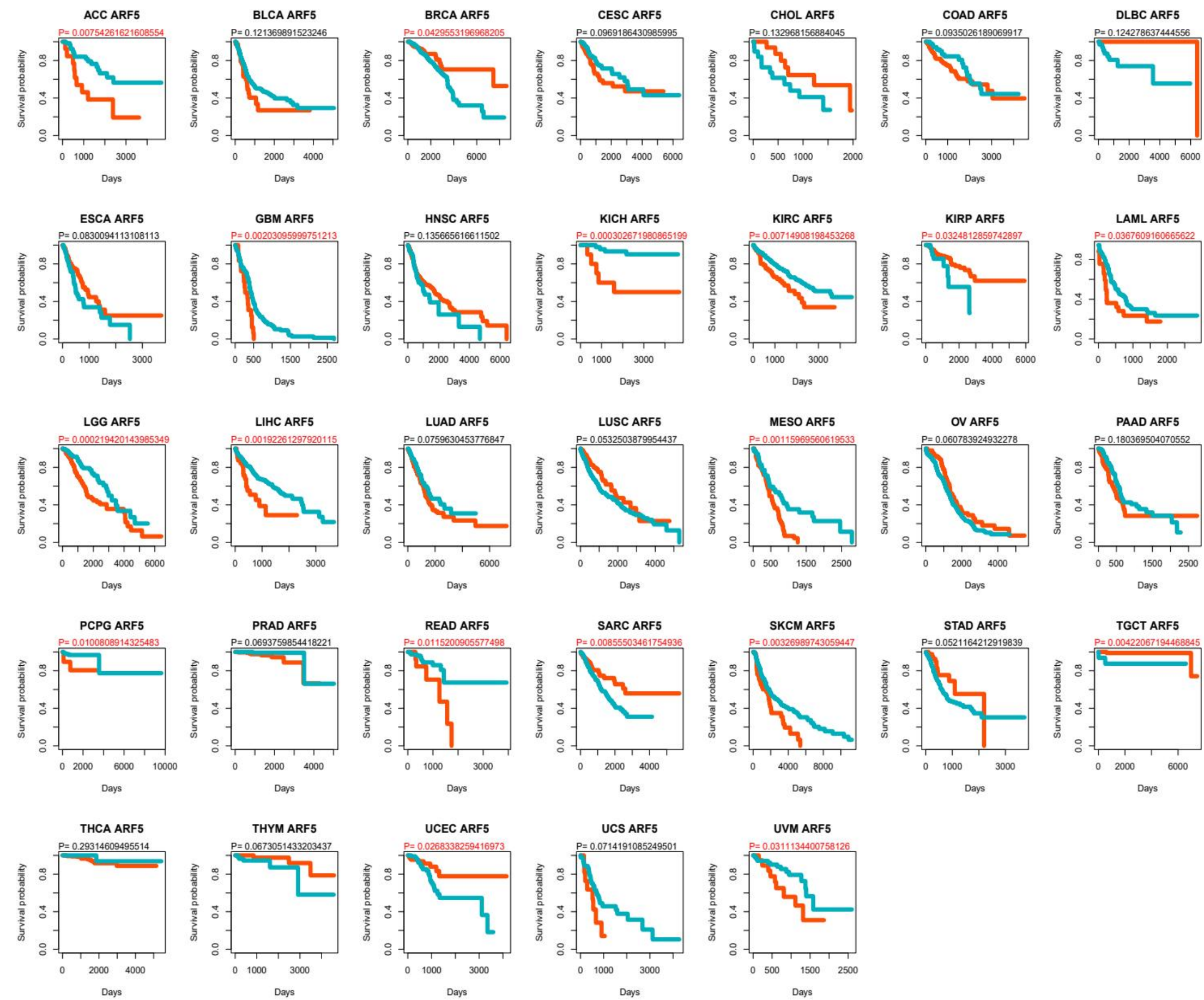**B****DFI of ARF5**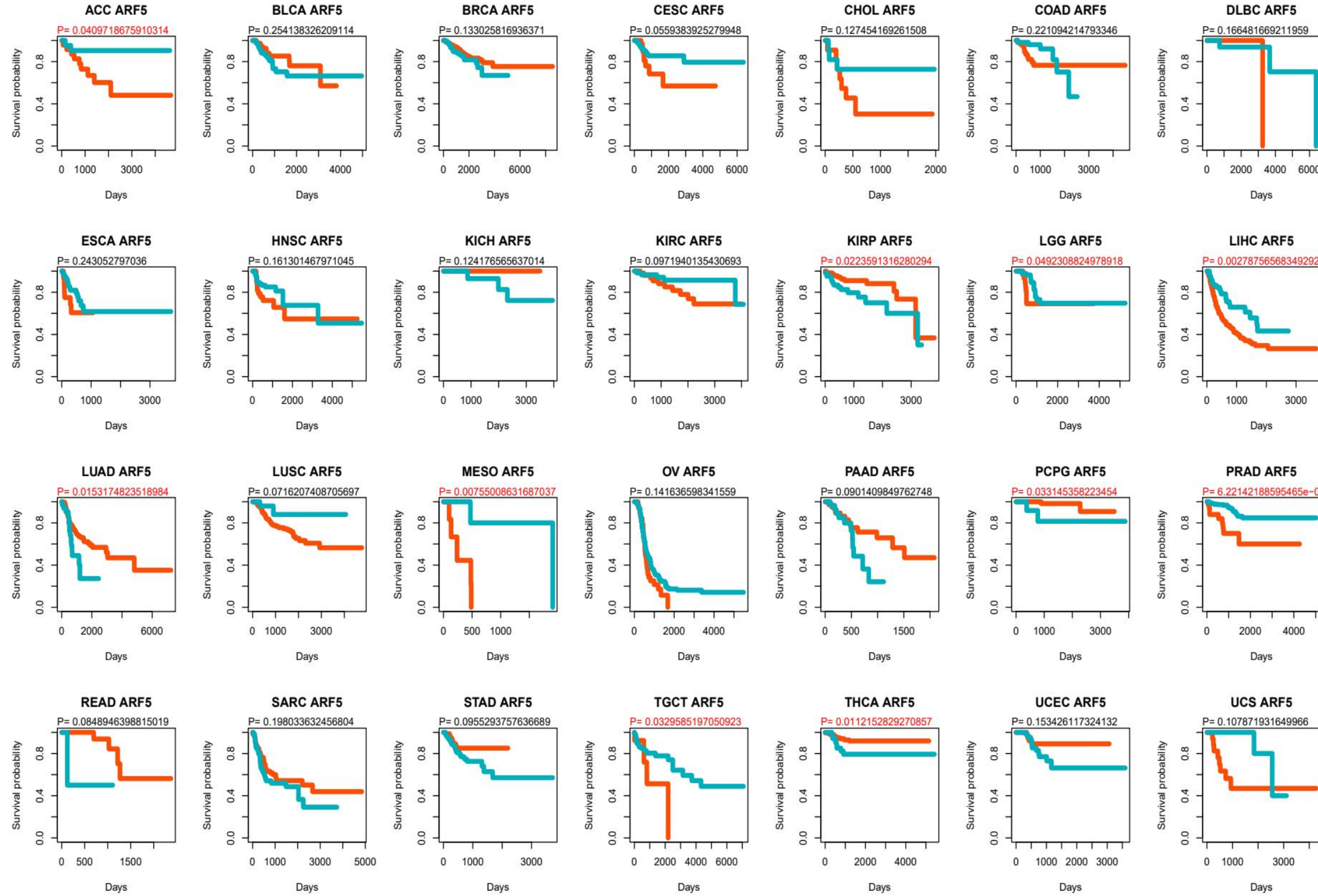**C****PFI of ARF5**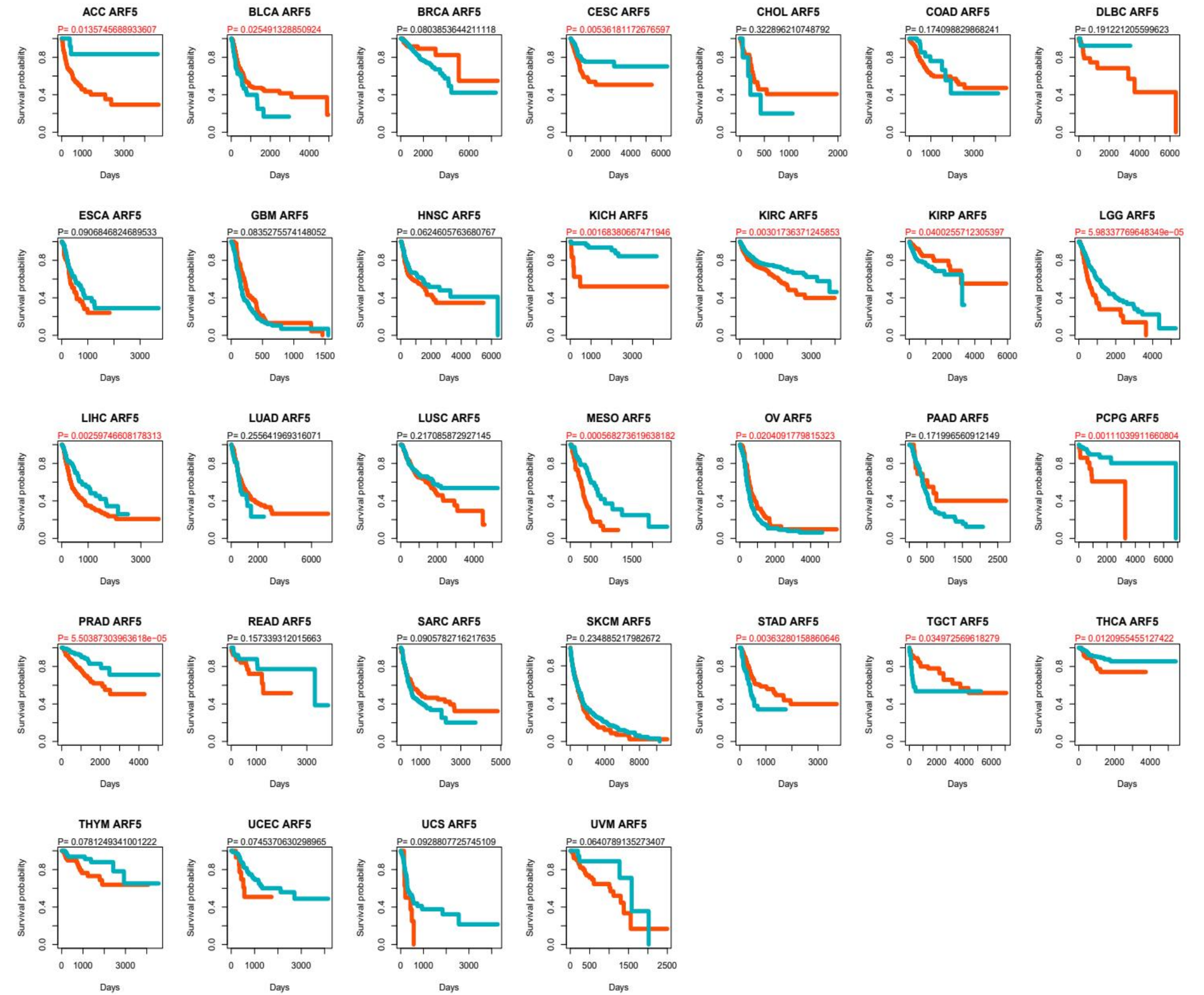**D****DSS of ARF5**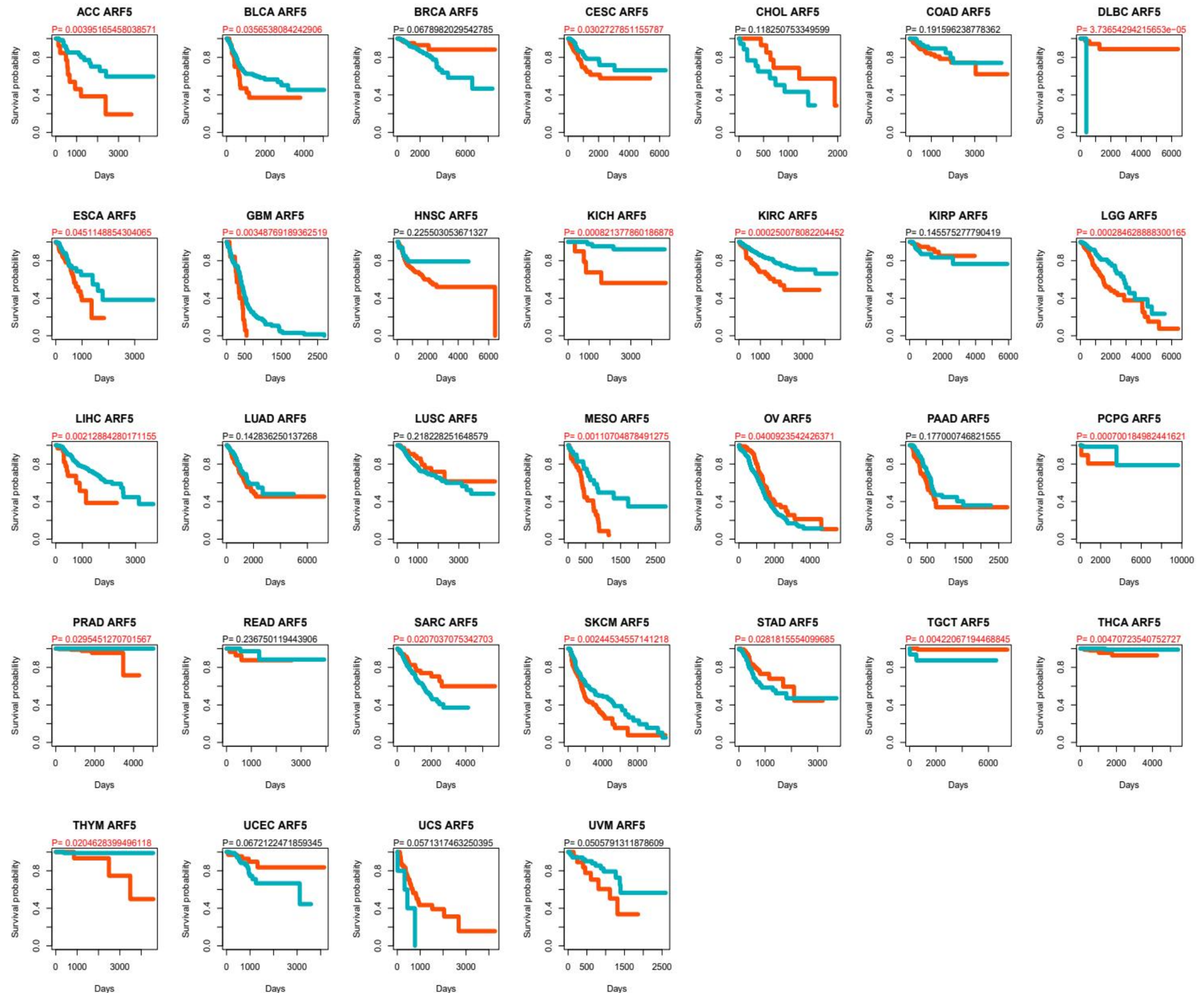

Supplement: Multimedia component 1 [file mmc1.pdf]

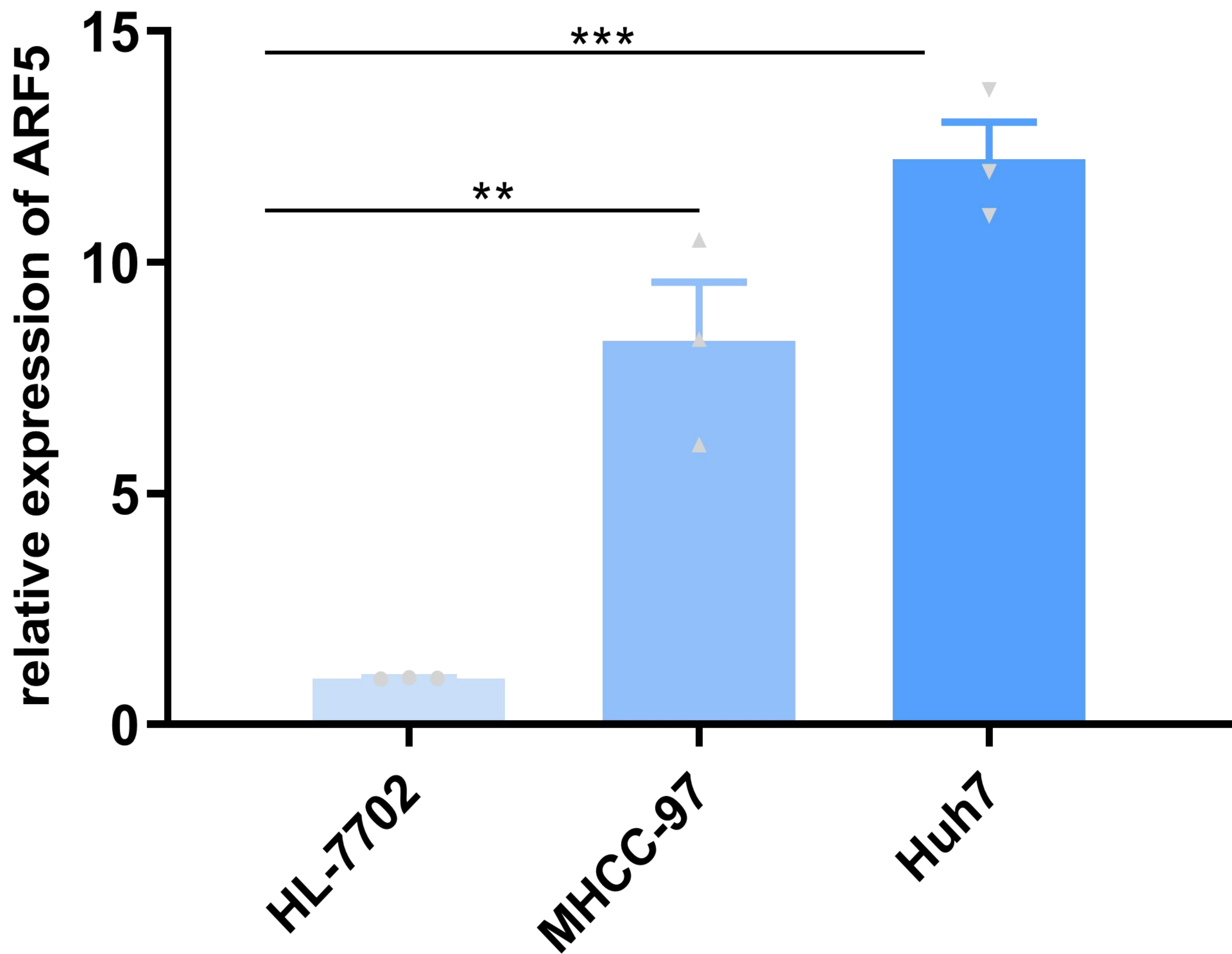

Supplement: Multimedia component 2 [file mmc2.pdf]

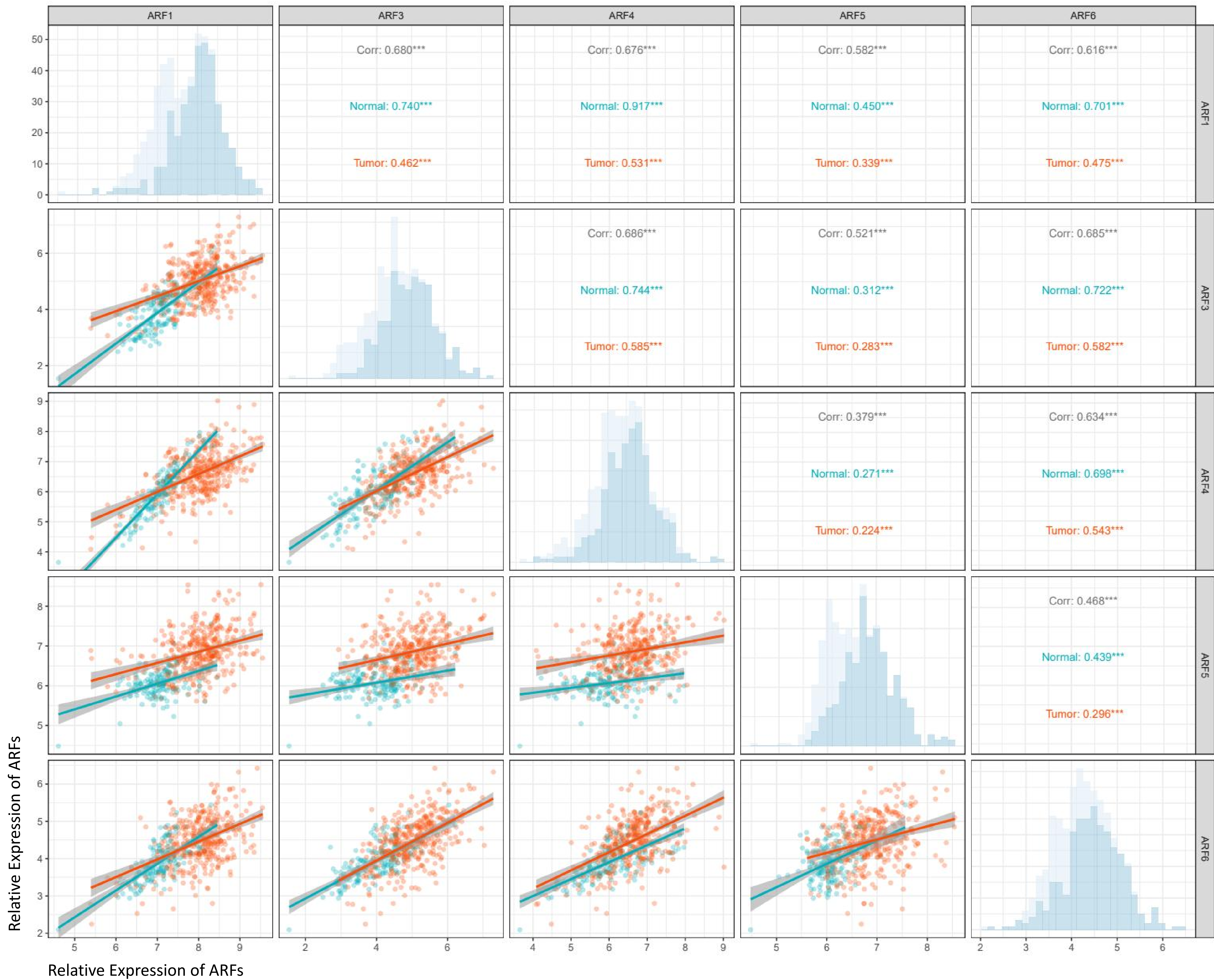

Supplement: Multimedia component 3 [file mmc3.pdf]

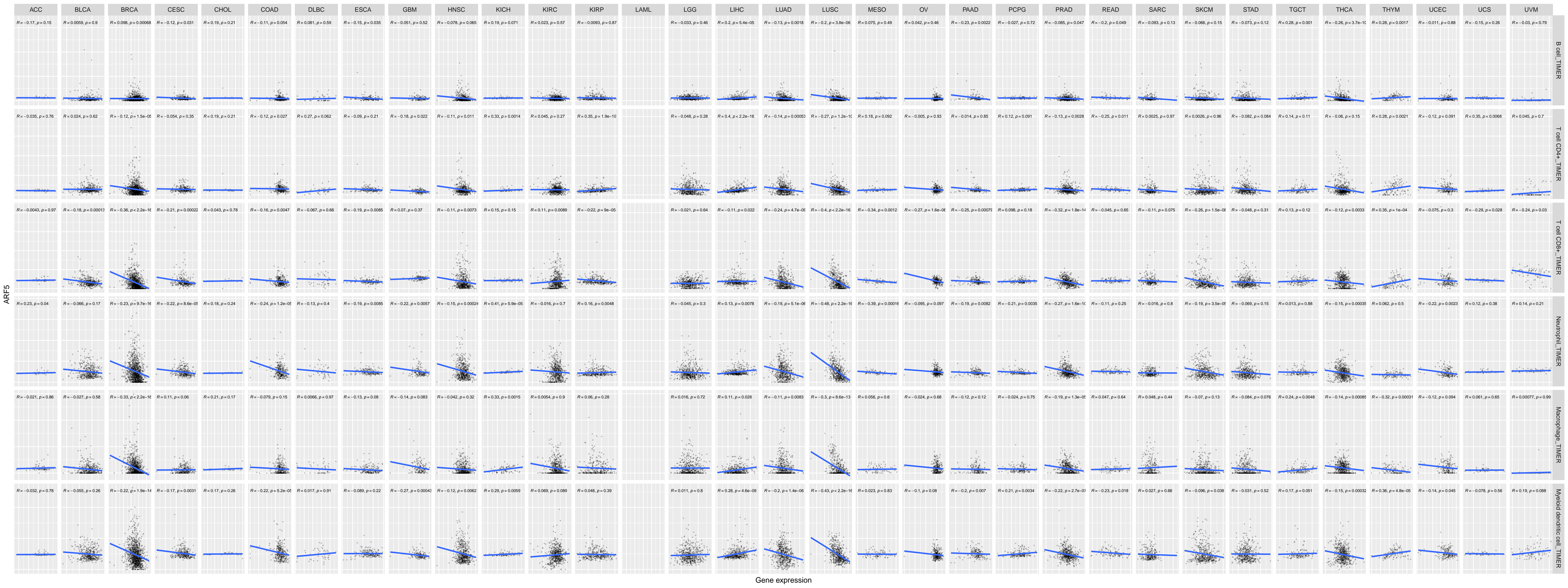

Supplement: Multimedia component 4 [file mmc4.pdf]
